# Enological Tannin Effect on Red Wine Color and Pigment Composition and Relevance of the Yeast Fermentation Products

**DOI:** 10.3390/molecules22122046

**Published:** 2017-11-23

**Authors:** Ignacio García-Estévez, Cristina Alcalde-Eon, Víctor Puente, M. Teresa Escribano-Bailón

**Affiliations:** 1 Grupo de Investigacion en Polifenoles, Departament of Analytical Chemistry, Nutrition and Food Science, Faculty of Pharmacy, University of Salamanca, Campus Miguel de Unamuno, E 37007 Salamanca, Spain; igarest@usal.es (I.G.-E.); crisalcaldeon@usal.es (C.A.-E.); 2 Laffort España, Polígono Txirrita Maleo 12, E 20100 Errenteria, Spain; victor.puente@laffort.com

**Keywords:** enological tannin, ellagitannin, anthocyanin, anthocyanin-derived pigments, vitisins, Tempranillo red wine, red wine color, CIELAB

## Abstract

Enological tannins are widely used in the winemaking process either to improve different wine characteristics (color stability, among others) or to compensate for low tannin levels. In this work, the influence of the addition of two different enological tannins, mainly composed of hydrolysable (ellagitannins) and condensed tannins, on the evolution of color and pigment composition of two different types of model systems containing the five main grape anthocyanins was studied. In addition, the effect of the addition of an enological tannin on the color and pigment composition of red wines made from *Vitis vinifera* L. cv Tempranillo grapes was also studied by high-performance liquid chromatography with diode array detection coupled to mass spectrometry (HPLC-DAD-MS). Results showed that, in model systems, the addition of the enological tannin favored the formation of anthocyanin-derived pigments, such as A-type and B-type vitisins and flavanol-anthocyanin condensation products, provided that the yeast precursors were previously supplied. Moreover, model systems containing the enological tannins were darker and showed higher values of chroma at the end of the study than control ones. The higher formation of these anthocyanin-derived pigments was also observed in the red wines containing the enological tannin. Moreover, these wine also showed lower lightness (L*) values and higher chroma (C*_ab_) values than control wines, indicating a higher stabilization of color.

## 1. Introduction

Wine color is one of the first features that consumers perceive. On the one hand, color can condition the acceptance of the wine. On the other, wine color provides important information about its age, quality, state of preservation, etc. Anthocyanins are the main compounds involved in the color of the wine. They are extracted from the grape skins to the must, being mainly responsible for the color of young wines. During wine maturation, anthocyanins progressively disappear because of various chemical reactions that lead to new derivatives. Anthocyanins and flavanols can condense either directly or by mediation of acetaldehyde or other compounds [1]. Some of these reactions cause bathochromical shifts in the visible absorption maxima of the anthocyanins, providing a bluish-red hue to the wine [2,3]. Other reactions causing anthocyanin transformation are those giving rise to pyranoanthocyanins. These anthocyanin-derived pigments result from the cycloaddition of wine nucleophiles at C4 and at the OH attached to C5 of the anthocyanin flavylium nucleus (followed by aromatization through autoxidation), which lead to an additional pyrane ring in the pigment structure [4]. Among the compounds present in wines, pyruvic acid [4,5], acetaldehyde [6,7], vinylphenol [5], vinylguaiacol [8,9], vinylcatechol [8,10], hydroxycinnamic acids [11], monomeric and dimeric procyanidins [12,13], acetone [7] and, more recently, acetoacetic acid and diacetyl [14], have been demonstrated to react with anthocyanins to generate these kinds of pigments. The cycloaddition reaction causes a hypsochromical shift in the visible absorption maxima of anthocyanins, thus producing a change in wine color towards orange hues [5,7,8,12,13].

The formation of all these anthocyanin-derived pigments is directly related to the presence in the wine of the different compounds involved in the reactions, i.e., the anthocyanins and these other compounds that become integrated in the final structure of the anthocyanin-derived pigments. The profiles of anthocyanins and flavanols depend on grape variety [15,16] and their levels can be conditioned by different factors, such as vintage or the moment of harvest. On the contrary, the levels of most of the precursors involved in the formation of pyranoanthocyanins depend on the type of yeast, and even on the yeast strain responsible for alcoholic fermentation [17]. Furthermore, other compounds present in wine, such as oak ellagitannins, seem to indirectly influence the formation of some of these anthocyanin-derived pigments. The oak ellagitannin structure allows them to participate in oxidation reactions acting as consumers of oxygen, protecting other compounds against oxidation and favoring the transformation of ethanol into acetaldehyde [18] which, in turn, might favor several polymerization reactions [18,19]. As a result, wine color can be modified by the presence of these oak wood compounds. The traditional source of ellagitannins in wine are the oak wooden barrels where the wine is kept during the aging process [20,21]. Nevertheless, they can also be released from the chips or staves that are used as an alternative to barrel aging to reduce costs. In addition, they can be supplied by the enological tannins that are used nowadays in the winemaking process either to improve different wine characteristics (color and stability among others) or to compensate for low tannin levels. There are a wide range of commercial tannins available, mostly composed of mixtures of condensed (proanthocyanidins) and hydrolyzable tannins (gallic and ellagic tannins) from different plant sources [22,23]. Some of the claims associated to their use in winemaking and aging include the protection of the wine against oxidation and the improvement of its flavor, mouthfeel, color, and stability, which might enhance the aging potential [22,24].

Regarding the potential benefits of the enological tannins on wine color, controversial results are reported. Whereas some studies reported a remarkable effect on the wine color intensity [24,25,26,27], others did observe little, or no, effects on wine color [28,29]. In previous studies carried out in our laboratory [30,31] we observed that enological tannins could affect anthocyanin and anthocyanin-derived pigments levels and, consequently, affect the final color of the wine. Differences between studies can be attributed to the fact that the grape varieties and the enological tannins employed in the different studies were not the same. Furthermore, there were also differences in the moment at which the enological tannin was added among the different studies. Since the grape variety can influence the effects caused by the enological tannins on color and pigment composition, it would be interesting to study these effects in simpler wine-like model systems than wine itself. In such model systems, the conditions can be set up to reduce the factors affecting the final effect of these enological tannins by reducing the side-reactions in which they might participate in wine. In addition, since the synthesis of some anthocyanin-derived pigments depends on the products of yeast metabolism and their production can be conditioned by the type and strain of yeast, it would also be interesting to control the supply of these precursors to the model systems. For this reason, the first objective of the present work was to study the evolution of the pigment profile and color in simple wine-like model systems that only contained the five main grape native anthocyanin 3-*O*-glucosides in the presence or in the absence of a standard medium resulting from the fermentative metabolism of glucose and either added, or not, with an enological tannin. Furthermore, in order to verify the results obtained in model systems, the evolution of the color parameters and anthocyanin and anthocyanin-derived pigments compositions of a red wine made from *Vitis vinifera* L. cv Tempranillo grapes in the presence of the same enological tannin were assessed.

## 2. Results and Discussion

### 2.1. Model Systems

#### 2.1.1. Evolution of Pigment Composition

**Figure 1:**
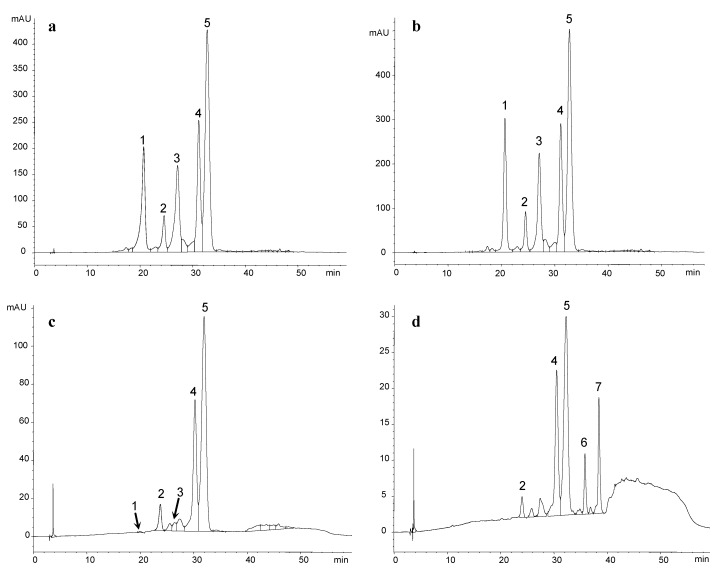
Chromatographic profile of model systems *A* (**a**,**c**) and *AF* (**b**,**d**) at day 0 (**a**,**b**) and at day 98 (**c**,**d**) recorded at 520 nm (each shown in full scale). Peaks 1 to 5: 3-*O*-glucosides of delphinidin, cyanidin, petunidin, peonidin, and malvidin, respectively. Peak 6, A and B-type vitisins of peonidin 3-*O*-glucoside. Peak 7, A and B-type vitisins of malvidin 3-*O*-glucoside.

The evolution of pigment composition in different model systems was monitored. They were prepared using two different solvents: standard wine (*A*, reference model system, and *AC* and *AS*, added with the tannin *C* and *S*, respectively) or a standard medium resulting from the fermentative metabolism of glucose (*AF*, reference model system, and *ACF* and *ASF*, added with the tannin *C* and *S*, respectively). In both types of model systems (in absence or presence of standard medium resulting from the fermentative metabolism of glucose), the disappearance of the anthocyanins over time was observed. The qualitative evolution of the pigments was the same for each type of model system regardless of the presence or absence of enological tannin and independently of the type of enological tannin employed. However, some important differences were observed depending on the solvent employed to prepare the model system. In the case of the model systems prepared with standard wine (Figure 1a,c for model system *A*) the disappearance of the grape anthocyanins (mainly delphinidin 3-*O*-glucoside and petunidin 3-*O*-glucoside, peaks 1 and 3 in Figure 1, respectively) was clearly observed, whereas no formation of anthocyanin-derived pigments could be observed. On the contrary, in the model systems prepared using the fermentative medium as solvent (Figure 1b,d, for model system *AF*), the disappearance of the grape anthocyanins occurred along with the formation of anthocyanin-derived pigments, as will be explained later.

**Figure 2:**
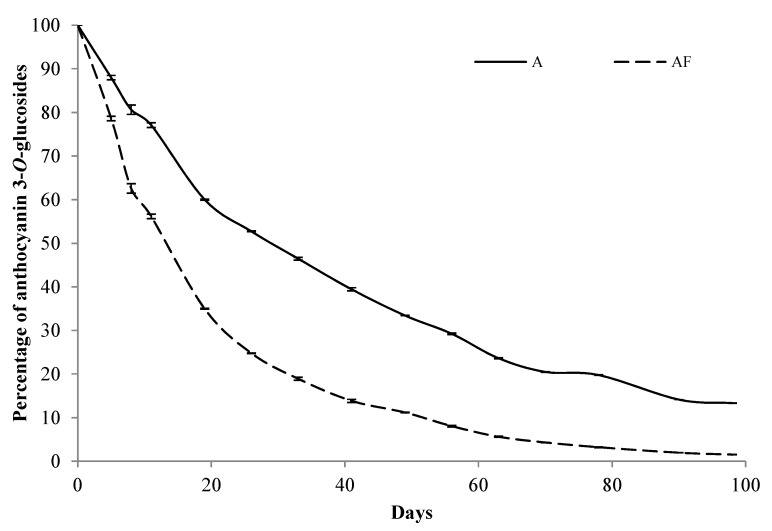
Evolution of the mean percentage (*n* = 6) of the total content of the anthocyanins in relation to their initial content in the model systems containing only anthocyanins in standard model wine (*A*) and in the solution resulting from fermentative metabolism of glucose (*AF*).

Quantitative differences were also observed between the two types of model systems. In the case of the model systems prepared with standard wine, the disappearance of the anthocyanins was slower and the final percentages of anthocyanins in relation to the initial concentration were higher than in the case of the model systems prepared in the fermentative medium (Figure 2). To be precise, whereas in the standard wine model systems the total anthocyanin content at the end of the study represented more than 10% of the initial content, in those prepared in the fermentative medium the final percentage was lower than 1%. The greater complexity of the fermentation medium in relation to the standard wine makes possible the involvement of anthocyanins in different reactions and, consequently, their transformation is more important than in standard wine model systems. In fact, as indicated above, the formation of anthocyanin-derived pigments was observed in the model systems prepared in the fermentative medium, namely the formation of A-type and B-type vitisins of peonidin 3-*O*-glucoside (peak 6 in Figure 1) and of malvidin 3-*O*-glucoside (peak 7 in Figure 1). The synthesis of these kinds of anthocyanin-derived pigments in the model systems prepared in the fermentative medium was possible since the precursors of these pigments (pyruvic acid for A-type vitisins and acetaldehyde for B-type vitisins) were available as a result of the fermentative metabolism of glucose.

Thus, the higher decrease in the levels of the anthocyanins in the fermentative medium was partly due to their involvement in the formation of anthocyanin-derived pigments, such as A-type and B-type vitisins. It is worth noting that, in both types of model systems, peonidin 3-*O*-glucoside was the anthocyanin that showed, at the end of the study, the higher percentages in relation to the initial contents. Moreover, A-type and B-type vitisins derived from this anthocyanin were also detected in the model systems prepared in the fermentative medium. Thus, the disappearance of this anthocyanin could be mainly related to the formation of anthocyanin-derived pigments. On the contrary, no derivative pigments from petunidin 3-*O*-glucoside or delphinidin 3-*O*-glucoside were detected in any of the studied model solutions although they were the anthocyanins showing the most important disappearance. This could be pointed out by a higher reactivity of these anthocyanins in relation to the others with probably faster degradation rates than the rest of the anthocyanins.

In both types of model systems, a small elevation of the baseline (hump) was observed at the end of the chromatograms of the latest sampling points, which might be related to the formation of oligomeric compounds, among others. However, there is not a tight relationship between the large decrease in the levels of the monoglucosides and the appearance of this hump. This indicated that most of the anthocyanin disappearance was due to the formation of non-colored compounds.

Regarding the influence of the presence of the enological tannin, in the case of the model systems prepared with standard wine, it was observed that model system *A* (control) showed the lowest disappearance of anthocyanins in relation to model systems *AC* and *AS* (see Tables S1 and S2 in the Supplementary Materials). However, in the case of the model systems prepared in the fermentative medium, the lowest loss of anthocyanins was observed for those model systems added with the enological tannins, and no differences were observed between the two different enological tannins. The higher complexity of the fermentative medium in relation to standard wine might have caused important differences on the redox status for the different type of model systems. Thus, the addition of the enological tannins can exert different effects depending on the solvent used.

**Figure 3:**
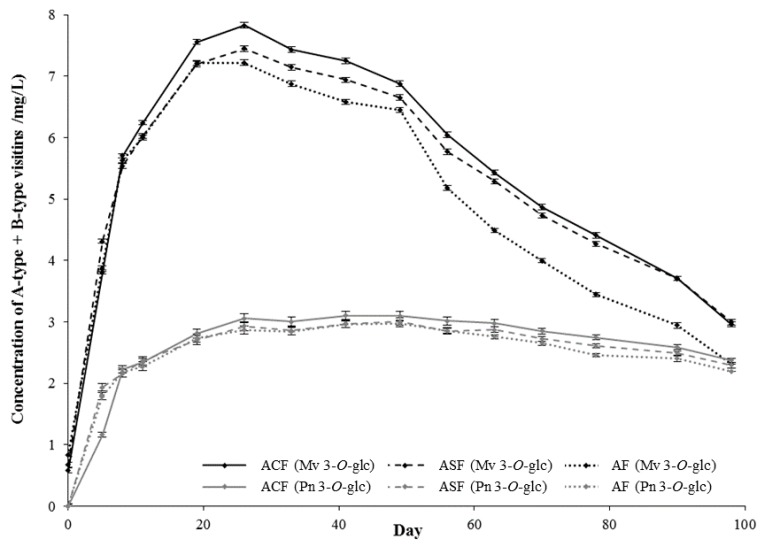
Evolution of the mean concentration (*n* = 6) of A-type + B-type vitisins of peonidin 3-*O*-glucoside (gray lines) and of malvidin 3-*O*-glucoside (black lines) in the model systems made with the standard medium resulting from the fermentative metabolism of glucose in the absence (*AF*) or presence of enological tannins (*ACF* and *ASF*, added with tannin *C* or *S*, respectively).

The formation of A-type and B-type vitisins, as explained above, was only observed in the model systems prepared in the fermentative medium. The formation of these anthocyanin-derived pigments in these model systems took place both in the presence and in the absence of enological tannin, although the presence affected their formation. From these results, it can be deduced that the formation of these pigments is mainly conditioned by the presence of pyruvic acid or acetaldehyde in the media. However, based on the absence of these anthocyanin-derived pigments in the models systems prepared in model-wine solution, it seems that the presence of the possible precursors (such as ethanol in the case of acetaldehyde) is not enough to allow their synthesis. With the chromatographic method employed in this study, the A-type and B-type vitisins of each anthocyanin co-eluted in the same peak in the chromatogram and for this reason they were quantified together. Figure 3 shows the evolution of the content of A-type + B-type vitisins of peonidin and malvidin 3-*O*-glucosides in the model systems prepared in the fermentative medium. The vitisins derived from malvidin 3-*O*-glucoside were detected in the first sampling point (2 h), whereas those derived from peonidin 3-*O*-glucoside were not detected until the second sampling point (five days). Furthermore, the levels of the former ones were higher than those of the latter ones during all the period studied which can be explained by the higher initial levels of malvidin 3-*O*-glucoside in relation to those of peonidin 3-*O*-glucoside. The model systems added with the enological tannins (*ACF* and *ASF*) showed the highest levels of vitisins, which might be related to the presence of ellagitannins in the enological tannins. On the one hand, they have been reported to favor the transformation of ethanol into acetaldehyde [18], one of the substrates for the formation of B-type vitisins. On the other hand, the ellagitannins present in the enological tannins can favor the formation of the A-type vitisins acting as oxidants in the last step of the synthesis, which is an essential step to complete it [32]. However, the absence of B-type vitisins in the model systems prepared in model-wine solutions (even in the presence of ellagitannins), along with the preliminary results of current studies carried out in our laboratory [33] where the formation of A- and B-type vitisins is studied in model-wine solution in the presence of individual ellagitannins and in the presence or absence of pyruvic acid, are indicating that the only presence of ethanol and ellagitannins does not lead to a significant formation of these anthocyanin-derived pigments. Thus, it seems that the main way in which ellagitannins could favor the formation of vitisins is related to their possible role as oxidants in the last oxidation step of the synthesis of these anthocyanin-derived pigments.

Moreover, flavanol-anthocyanin acetaldehyde-mediated condensation products were also detected (but could not be quantified due to their low levels) in the model system *ACF*. The higher levels of proanthocyanidins in the enological tannin *C* in relation to the enological tannin *S* could explain why these anthocyanin-derived pigments were only detected in those model systems added with enological tannin *C*.

#### 2.1.2. Color Evolution

The evolution of the CIELAB color parameters calculated from the visible spectra of the model systems at each sampling point was studied. Regarding lightness (L*, Table 1), it increased for all the samples over time, which was in accordance with the decrease observed in the total pigment content [34]. Nevertheless, the magnitude of the increase was different for the different model systems. Thus, at the end of the study and in relation to the initial value, it was observed an increase of ca. three units for the standard wine-model systems and of ca. 12 units for the model systems prepared in the fermentative medium. The difference on the evolution of the lightness could be attributed to the difference on the evolution of the pigment composition [34] since, in the model systems *ACF*, *ASF*, and *AF*, the loss of anthocyanins was much higher than in *AC*, *AS*, and *A*.

For a same type of model system (model wine or fermentative medium), the lowest values of lightness were observed in those added with the enological tannins. The enological tannins employed in the present study contained, among other compounds, proanthocyanidins and hydroxybenzoic acids, which may act as co-pigments of the anthocyanins [35]. Consequently, the lightness of the samples could be reduced due to the hyperchromic effect induced by co-pigmentation [35]. No differences were observed between the lightness values of the model systems depending on the type of enological tannin added and, in both cases, the model system were darker than the corresponding model system without enological tannin.

The evolution of chroma (C*_ab_) was similar for all the model systems studied (Table 2). In all cases a decrease in the values of chroma was observed over time, which can be attributed to the loss of pigments [34]. However, as in the case of L*, the change observed in the values of C*_ab_ of the model systems prepared in the fermentative medium was more important than that observed for the model systems prepared with standard wine. It can be related to the lower loss of anthocyanins in the latter ones. The model systems treated with enological tannins were those that showed the lowest decreases in the value of chroma and, consequently, were those that showed the highest values at the end of the study. As in the case of lightness, the difference in the chroma value between model systems *A* and *AC* and *AS* might be related to differences on the co-pigmentation phenomenon [35].

The hue was affected by the addition of the enological tannins in the same way in both types of model systems (Table 3). The model systems to which the enological tannin was not added (*A* and *AF*) showed lower values of hue than those to which either the enological tannin *C* (*AC* and *ACF*) or the enological tannin *S* (*AS* and *ASF*) was added. In the case of model systems prepared in the fermentative medium, it could be explained by the higher formation of anthocyanin-derived pigments such as A-type and B-type vitisins, which show an orange-red color [34], i.e., higher hue values than the corresponding anthocyanins. In the case of model systems prepared with standard wine, the lower hue values observed in model system *A* could be attributed to the higher levels of anthocyanins in this media in relation to model systems *AC* and *AS*. Moreover, it has to be pointed out that enological tannins showed, when solubilized, a light yellow-brown color that could affect, mainly, the hue of the model systems. Thus, the highest values of hue observed in the model systems added with enological tannins might also be explained by the contribution to color of the enological tannins.

### 2.2. Wines

#### 2.2.1. Evolution of Pigment Composition

The results observed in the model systems pointed out that the differences on the evolution of pigment composition were mainly related to the composition of the solvent used in the different model solutions. Since those differences are due to the use of a solution resulting from the fermentative metabolism of glucose, it should be interesting to study the effect of adding an enological tannin during winemaking. Thus, the evolution of wine pigment composition was studied for eight months at five different points of the winemaking and aging processes in two different wines (made in triplicate): control wine (Wine *C*), with no addition of any enological tannin, and Wine *R*, added with the enological tannin (tannin *C*), and fermented with the same strain of *S. cerevisiae* than Wine *C*. Table 4 shows the evolution of pigment composition. At the end of the alcoholic fermentation (17 days) wines *R* showed higher levels of anthocyanins than Wine *C*. Thus, it seems that the addition of proanthocyanidins and phenolic acids through the addition of the enological tannin can favor the extraction of anthocyanins from grapes by means of co-pigmentation reactions as previously reported elsewhere [35,36,37]. These results are in accordance with previous studies performed in our laboratory in which it was reported that the addition of an enological tannin containing both condensed and hydrolyzable tannins could increase the levels of anthocyanins in wines and stabilize wine color through co-pigmentantion reactions [30,31].

Furthermore, the wine containing the enological tannin also showed, at the end of the study, a lower loss of anthocyanins than Wine *C*. To be precise, the levels of these pigments decreased ca. 40 mg/L less in wine *R* than in Wine *C*. This could be related to a higher protection of these pigments against oxidation as a consequence of the presence of ellagitannins in these wines [18] due to the addition of the enological tannin. This observation was in accordance with the results obtained in the model systems prepared in the fermentative medium since, in these model systems, the highest levels of anthocyanins corresponded to those added with an enological tannin.

Several differences were also observed in relation to the anthocyanin-derived pigments (Table 4). Respecting the flavanol-anthocyanin direct condensation products (F-A^+^ products) the highest levels were found in Wine *R*. In literature, it has been proposed that the presence of ellagitannins might favor the polymerization reactions between flavanol and anthocyanins [18,38]. In fact, previous results obtained in our laboratory pointed out that the use of an enological tannin during the winemaking of commercial wines can favor the formation of this kind of pigments [30,31]. Thus, the addition of the enological tannin could not only supply substrates (flavanols), but also indirectly favor the formation of this kind of pigments.

In the case of the flavanol-anthocyanin acetaldehyde-mediated condensation products, Wine *R* also showed higher levels than Wine *C*. The presence of ellagitannins, which are related to a higher formation of acetaldehyde [18] and the higher levels of proanthocyanidins in Wine *R*, as a consequence of the addition of the enological tannin, could explain the higher formation of this kind of pigments.

The formation of A-type and B-type vitisins of malvidin 3-*O*-glucoside was also affected by the addition of the enological tannins. Wine *R* showed higher levels of these vitisins than Wine *C*. Thus, it seems that the addition of the enological tannin could also favor the formation of these vitisins in red wines. As previously indicated for the model systems, the higher formation of this anthocyanin-derived pigments can be attributed to the presence of ellagitannins, which might take part in the oxidation step required to complete the synthesis of A-type vitisins [32]. In fact, previous studies [30,31] showed that the addition of enological tannins containing ellagitannins favored the formation of vitisins in wine, since the levels determined in those treated wines were higher than those observed in control wines without the addition of enological tannins.

Finally, the formation of the vinylphenol-type pyranoanthocyanin of malvidin 3-*O*-glucoside was not affected by the addition of the enological tannin. This is in accordance with the results obtained when an enological tannin was added during the winemaking of commercial wines, which also showed that the levels of this kind of pyranoanthocyanins were not affected by the addition of enological tannins [30]. Thus, unlike in the case of A-type and B-type vitisins, the presence of ellagitannins seems not to affect to the formation of this kind of pyranoanthocyanin.

#### 2.2.2. Color Evolution

Table 5 shows the evolution of the CIELAB color parameters of the different wines during winemaking and aging. As observed in the model systems, the addition of the enological tannins led to wines with lower values of lightness and higher values of chroma. Thus, wine *R* showed during all the study, the lowest values of L*. This could be related to the higher levels of anthocyanins observed in this wine in relation to wine *C*. Moreover, the increase of the L* value over time in wine *C* is higher than in Wine *R*, probably as a result of the more important loss of anthocyanins in the former one.

Wine *R* also showed higher values and a lower decrease of C*_ab_ than Wine *C*, which can also be attributed to the higher levels of anthocyanins in the former. Thus, the wine to which the enological tannin was added was darker and with higher chroma than the control wine.

As for hue, it was observed that although there were small differences at initial steps between both types of wine, at the end of the study the wine added with the enological tannin showed lower values of hue than Wine *C*. Thus, the effect of the enological tannin could be mainly noticed during the aging process. These differences in the hue between the wine treated with enological tannin and the control one could be attributed to the protection of the anthocyanins against oxidation due to the presence of phenolic compounds, among them, ellagitannins. Thus, in Wine *C*, the oxidation of pigments, and consequently the formation of brown pigments during aging, seemed to be more important than in Wine *R*, and for this reason the hue of the former is higher (less red and more orange).

## 3. Material and Methods

### 3.1. Chemicals

The 3-*O*-monoglucosides of delphinidin, cyanidin, petunidin, peonidin, and malvidin were extracted from the skins of *Vitis vinifera* L. cv. Tempranillo grapes following the procedure previously used for delphinidin 3-*O*-glucoside isolation [39]. Briefly, extraction was carried out using MeOH/aqueous HCl 12 M (999:1). Methanol was evaporated under reduced pressure and the residue was re-dissolved in aqueous HCl (0.1 M, pH 1) and then loaded on a Sephadex LH-20 (Sigma-Aldrich, St. Louis, MO, USA) column (30 mm × 300 mm), which was previously conditioned using 1 L of aqueous HCl (0.1 M, pH 1). Elution was carried out using the same aqueous HCl solution and fractions (25 mL each) were collected. Anthocyanin monoglucosides eluted in the first fractions (malvidin, peonidin, petunidin, cyanidin and delphinidin 3-*O*-glucosides, cited in elution order) before the elution of the acylated anthocyanidins. The fractions containing the monoglucosides were gathered and then freeze-dried to furnish a reddish-purple powder.

The enological tannins were provided as a powder by Laffort España (Rentería, Spain). Two types of tannins (tannins *C* and *S*) were assayed. According to the manufacturer, tannin *C* can be added to the wine to improve the stability of the colouring matter, whereas the addition of tannin *S* is intended to improve its overall sensorial quality. A solution of each enological tannin (0.2 mg/mL) was analyzed by means of HPLC-DAD-MS according to García-Marino and co-workers [40] in order to assess their phenolic composition. The results showed that enological tannin *C* was mainly constituted by catechins (25.7%), dimeric (11.7%), and trimeric (4.6%) procyanidins, gallocatechins and prodelphinidins (16.8%), oak ellagitannins (11.2%), and hydroxybenzoic acids (7.5%). Enological tannin *S*, in turn, was mainly constituted by procyanidins (22.9%), catechins (8.4%), oak ellagitannins (16.9%), and hydroxybenzoic acids (11.1%).

### 3.2. Model Systems

The model systems (50 mL) consisted of a 3:1 mixture of the main grape native anthocyanidin (delphinidin, cyanidin, petunidin, peonidin, and malvidin) 3-*O*-glucosides (300 mg/L) with the enological tannin (*C* or *S*). They were prepared using two different solvents: standard wine (model systems *AC* and *AS*, added with the tannin *C* and *S*, respectively) or a standard medium resulting from the fermentative metabolism of glucose (model systems *ACF* and *ASF*, added with the tannin *C* and *S*, respectively). Model systems composed of a solution of the main anthocyanins at the same concentration and without enological tannins in both solvents (model systems *A* and *AF* in standard wine and in the fermentative medium, respectively) were used as a reference. Two model systems of each type were prepared and monitored. Analyses were performed in triplicate.

Standard wine consisted of 10% (*v*/*v*) ethanol in water containing 0.5% of tartaric acid (*w*/*v*) adjusted to pH 3.6 with NaOH 0.1 N. The solution resulting from the fermentative metabolism of glucose was obtained following the method described by Ough and co-workers [41]. Briefly, a synthetic must (200 g/L sugar, 300 mg/L total yeast assimilable nitrogen (YAN)) was fermented by *Saccharomyces cerevisiae* up to a residual concentration of 2 g/L of sugars (9.15% of ethanol, pH 3.2) and then it was centrifuged in order to separate the yeast cells from the liquid solution.

These model systems were stored in the dark under controlled temperature without shaking. They were monitored by means of HPLC-DAD-MS*^n^* analysis to study their pigment compositions and to evaluate their evolutions. Colorimetric measurements were also performed. Model systems were sampled until the disappearance of total anthocyanin was higher than 95% (98 days). Sampling was done the first day, twice each week during the first month, and weekly during the last two months.

### 3.3. Wines

Wines were made from *Vitis vinifera* L. cv. Tempranillo grapes collected from a vineyard located at D.O. Rioja at technological maturity (24 °Brix) fermented with *S. cerevisiae* (*Zymaflore RJA64*, Laffort España, Rentería, Spain). The enological tannin *C* was added at three steps (to a final concentration of 35 g/hL) as the manufacturer recommends the control wine (wine *C*) be made under the same conditions, but without any enological tannin. Fermentation was carried out in stainless steel fermenters (100 L) under winery conditions. For further details about the characteristics of grapes and the winemaking process see Supplementary Materials. Each type of wine was made in triplicate (*n* = 3). Wines have been monitored for five months by means of HPLC-DAD-MS*^n^* analysis and colorimetric measurements were also performed. Samples were taken at the end of alcoholic fermentation (17 days), after post-fermentative maceration (20 days), after malolactic fermentation (MLF) (two months), three months after the end of MLF (five months) and before bottling (eight months). Wine samples were diluted (1/5) with acidified water (pH 1, HCl) and filtered (0.45 μm) prior to HPLC-DAD-MS*^n^* analyses. For the colorimetric measurements, the pH of the wines was adjusted to 3.6 with a minimum volume of 3 N HCl to avoid dilution of the samples.

### 3.4. HPLC-DAD-MS Analysis

Analyses of anthocyanins and anthocyanin-derived pigments were performed using a Hewlett-Packard 1100 series liquid chromatograph (Agilent Technologies, Waldbronn, Germany). An AQUA C18 reversed-phase, 5 μm, 150 mm × 4.6 mm column (Phenomenex^®^, Torrance, CA, USA) thermostatted at 35 °C was used. The HPLC-DAD conditions were optimized for the analysis of anthocyanins. The solvents employed were: (A) an aqueous solution (0.1%) of trifluoroacetic acid; (B) 100% HPLC-grade methanol (HiPerSolv^®^ Chromanorm, BDH Prolabo, VWR International, Briare, France), establishing the following gradient: from 15 to 25% B for 10 min, from 25 to 35% B for 23 min, from 35 to 38% B for 15 min and isocratic 38% B for 2 min, at a flow rate of 0.6 mL/min. Detection was carried out at 520 nm as preferred wavelength. Spectra were recorded from 220 to 600 nm. Mass spectrometry was carried out using a Finnigan^TM^ LCQ ion trap instrument (Thermoquest, San Jose, CA, USA) equipped with an electrospray ionisation (ESI) interface. The LC system was connected to the probe of the mass spectrometer via the UV cell outlet. Both the sheath gas and the auxiliary gas were nitrogen. The sheath gas flow was 1.2 L/min and the auxiliary gas flow, 6 L/min. Spectra were recorded in positive ion mode between *m*/*z* 120 and *m*/*z* 1500. The mass spectrometer was programmed to perform a series of three consecutive scans: a full mass, a MS^2^ scan of the most abundant ion in the full mass and an MS^3^ of the most abundant ion in the MS^2^. The normalized energy of collision was 35%. Samples were filtered through a 0.45 μm Millex syringe-driven filter unit (Millipore Corporation, Bedford, MA, USA) before HPLC-DAD-MS analysis.

Identification of anthocyanins and derived pigments was carried out from the chromatographic (retention time), spectral features (UV-VIS spectra, *m*/*z* ratio of the molecular ions and fragmentation pattern in MS^2^ and MS^3^ analyses), and by comparison to previous reported data [38]. Quantification was performed from the area values of the chromatographic peaks and expressed as malvidin 3-*O*-glucoside equivalents (mg/L) except for vitisins in model systems, which were expressed as A-type vitisin of malvidin 3-*O*-glucoside (mg/L).

### 3.5. Colorimetric Measurements

Absorption spectra (190–1100 nm) were recorded using a Hewlett-Packard UV-VIS HP3853 spectrophotometer (Agilent Technologies, Waldbronn, Germany) in 2 mm path length quartz cells. The analysis of color was made only from the visible spectra (380–770 nm) data, using the CIE 1964 standard observer (10° visual field) and the CIE standard illuminant D65 as references. CIELAB color parameters (L*, a*, b*, C*_ab_, and h_ab_) were calculated using the software Cromalab^TM^ (University of Sevilla, Sevilla, Spain). Color differences (ΔE*_ab_) were also calculated using the equation ΔE*_ab_ = [(ΔL*)^2^ + (Δa*)^2^ + (Δb*)^2^]^1/2^ where ΔL*, Δa*, and Δb* are the differences between the CIELAB parameters. Samples were measured in triplicate.

### 3.6. Statistical Analysis

Statistical differences were determined by one-way analysis of variance (ANOVA) and Tukey’s Honestly Significant Differences (HSD) test. The IBM SPSS Statistics 21 for Windows software package (IBM, Armonk, NY, USA) was used for data processing.

## 4. Conclusions

The effect of the addition of enological tannins mainly composed of hydrolyzable and condensed tannins on the color and pigment evolution of model systems containing the five main grape anthocyanins and red wines was assessed. As for model systems, important differences were observed depending on the solvent employed. The highest anthocyanin transformation was observed in those model systems prepared using the fermentative medium. In this case, the model systems containing the enological tannins showed, at the end of the study, the highest levels of anthocyanins, as well as the highest levels of A-type and B-type vitisins and of flavanol-anthocyanin direct condensation products. Moreover, these model systems showed the lowest lightness values and the highest chroma values at the end of the study. The results obtained in wines confirmed those obtained in model systems. Thus, a higher formation of anthocyanin-derived pigments, such as A-type and B-type vitisins or flavanol-anthocyanin acetaldehyde-mediated condensation products, was observed in the wines supplied with enological tannins. Furthermore, it seems that the addition of the enological tannin protects the anthocyanins against oxidation, which might be also related to the presence of ellagitannins in the enological tannin. Wines treated with the enological tannin were darker red wines (lower L*) and showed higher values of chroma at the end of the study, which could indicate a higher stabilization of color. All these results highlight the relevance of the products of yeast fermentation in the evolution of the anthocyanin and anthocyanin-derived pigments and the role of the enological tannins in favoring the synthesis of the derivatives related to yeast fermentation products. Thus, an adequate selection of the yeast strain combined with the use of enological tannins containing a mixture of phenolic compounds can be employed to direct the transformation of the grape anthocyanins to certain derivatives, which are, some of them, more stable from a chemical point of view.

## Figures and Tables

**Table 1:** Evolution of the mean value of lightness (L*) in the model systems.

Day	Standard Wine ^1^			Fermentative Medium ^1^		
	AC	AS	A	ACF	ASF	AF
0	86.97 ± 0.08 ^a^	86.64 ± 0.09 ^a^	87.18 ± 0.08 ^a^	77.97 ± 0.08 ^a^	78.62 ± 0.06 ^a^	78.35 ± 0.09 ^a^
5	86.75 ± 0.03 ^a^	86.48 ± 0.05 ^a^	87.05 ± 0.04 ^b^	79.25 ± 0.08 ^a^	79.79 ± 0.08 ^b^	79.99 ± 0.02 ^c^
19	88.33 ± 0.06 ^a^	87.85 ± 0.01 ^b^	88.68 ± 0.03 ^c^	83.35 ± 0.04 ^a^	83.71 ± 0.02 ^b^	84.31 ± 0.02 ^c^
41	89.74 ± 0.05 ^a^	89.24 ± 0.02 ^b^	90.24 ± 0.05 ^c^	85.91 ± 0.05 ^a^	86.25 ± 0.02 ^b^	87.26 ± 0.06 ^c^
70	88.50 ± 0.04 ^a^	88.18 ± 0.01 ^b^	90.08 ± 0.02 ^c^	89.51 ± 0.06 ^a^	89.54 ± 0.04 ^a^	90.58 ± 0.07 ^b^
90	88.95 ± 0.03 ^a^	88.86 ± 0.02 ^b^	90.87 ± 0.03 ^c^	89.97 ± 0.01 ^a^	89.99 ± 0.04 ^a^	90.99 ± 0.02 ^c^
98	89.02 ± 0.01 ^a^	89.26 ± 0.07 ^b^	91.10 ± 0.03 ^c^	90.32 ± 0.02 ^a^	90.44 ± 0.01 ^b^	91.41 ± 0.05 ^c^

^1^ *AC*: Model system in standard wine added with tannin *C*. *AS*: Model system in standard wine added with tannin *S*. *A*: Reference model system in standard wine. *ACF*: Model system in the fermentative medium added with tannin *C*. *ASF*: Model system in the fermentative medium added with tannin *S*. *AF*: Reference model system in the fermentative medium. Different lower case letters within each row and for each type of medium (standard wine or fermentative medium) indicate significant differences (*n* = 6, *p* < 0.05).

**Table 2:** Evolution of the mean value of chroma (C*_ab_) in the model systems.

Day	Standard Wine ^1^			Fermentative Medium ^1^		
	AC	AS	A	ACF	ASF	AF
0	22.65 ± 0.06 ^a^	22.90 ± 0.08 ^a^	21.42 ± 0.08 ^a^	40.71 ± 0.09 ^a^	39.54 ± 0.06 ^a^	40.40 ± 0.07 ^b^
5	21.30 ± 0.04 ^a^	21.52 ± 0.03 ^a^	21.09 ± 0.02 ^b^	36.35 ± 0.04 ^a^	35.24 ± 0.05 ^b^	35.60 ± 0.05 ^c^
19	17.02 ± 0.02 ^a^	17.44 ± 0.01 ^a^	16.93 ± 0.03 ^b^	24.26 ± 0.06 ^a^	23.53 ± 0.05 ^b^	22.98 ± 0.03 ^c^
41	13.56 ± 0.05 ^a^	13.92 ± 0.05 ^b^	13.02 ± 0.01 ^c^	17.21 ± 0.03 ^a^	16.37 ± 0.03 ^b^	15.39 ± 0.08 ^c^
70	13.23 ± 0.06 ^a^	12.90 ± 0.02 ^b^	11.01 ± 0.02 ^c^	12.75 ± 0.04 ^a^	12.11 ± 0.03 ^a^	10.88 ± 0.05 ^b^
90	12.36 ± 0.02 ^a^	11.91 ± 0.03 ^b^	9.69 ± 0.04 ^c^	11.98 ± 0.03 ^a^	11.37 ± 0.02 ^a^	9.96 ± 0.02 ^b^
98	12.24 ± 0.02 ^a^	11.41 ± 0.04 ^b^	9.35 ± 0.05 ^c^	11.68 ± 0.01 ^a^	10.93 ± 0.04 ^b^	9.55 ± 0.01 ^c^

^1^ *AC*: Model system in standard wine added with tannin *C*. *AS*: Model system in standard wine added with tannin *S*. *A*: Reference model system in standard wine. *ACF*: Model system in the fermentative medium added with tannin *C*. *ASF*: Model system in the fermentative medium added with tannin *S*. *AF*: Reference model system in the fermentative medium. Different lower case letters within each row and for each type of medium (standard wine or fermentative medium) indicate significant differences (*n* = 6, *p* < 0.05).

**Table 3:** Evolution of the mean value of hue (h_ab_) in the model systems.

Day	Standard Wine ^1^			Fermentative Medium ^1^		
	AC	AS	A	ACF	ASF	AF
0	−3.69 ± 0.09 ^a^	−3.20 ± 0.08 ^b^	−4.22 ± 0.01 ^c^	−1.36 ± 0.08 ^a^	−1.35 ± 0.06 ^a^	−1.25 ± 0.03 ^b^
5	2.10 ± 0.03 ^a^	2.63 ± 0.04 ^b^	1.32 ± 0.04 ^c^	0.51 ± 0.08 ^a^	0.59 ± 0.01 ^a^	0.16 ± 0.01 ^b^
19	14.16 ± 0.03 ^a^	13.46 ± 0.03 ^b^	10.32 ± 0.05 ^c^	7.80 ± 0.04 ^a^	6.74 ± 0.02 ^b^	6.42 ± 0.07 ^c^
41	26.73 ± 0.02 ^a^	24.05 ± 0.01 ^b^	18.57 ± 0.08 ^c^	21.58 ± 0.03 ^a^	18.36 ± 0.02 ^b^	15.84 ± 0.06 ^c^
70	33.83 ± 0.05 ^a^	30.21 ± 0.08 ^b^	28.12 ± 0.02 ^c^	37.02 ± 0.07 ^a^	34.44 ± 0.07 ^a^	28.80 ± 0.07 ^b^
90	41.34 ± 0.05 ^a^	37.49 ± 0.03 ^b^	36.23 ± 0.01 ^c^	43.17 ± 0.04 ^a^	39.16 ± 0.04 ^a^	34.57 ± 0.02 ^c^
98	43.23 ± 0.01 ^a^	39.28 ± 0.02 ^b^	38.50 ± 0.03 ^c^	44.72 ± 0.02 ^a^	40.76 ± 0.04 ^b^	36.16 ± 0.01 ^c^

^1^ *AC*: Model system in standard wine added with tannin *C*. *AS*: Model system in standard wine added with tannin *S*. *A*: Reference model system in standard wine. *ACF*: Model system in the fermentative medium added with tannin *C*. *ASF*: Model system in the fermentative medium added with tannin *S*. *AF*: Reference model system in the fermentative medium. Different lower case letters within each row and for each type of medium (standard wine or fermentative medium) indicate significant differences (*n* = 6, *p* < 0.05).

**Table 4:** Evolution of the pigment composition in wines *C* and *R* (mg/L, quantified as equivalents of malvidin 3-*O*-glucoside).

		Sampling Point ^1^				
		17 Days	20 Days	2 Months	5 Months	8 Months
Total anthocyanins	Wine *C*	380 ± 4 ^a^	319 ± 8 ^a^	281 ± 3 ^a^	264 ± 7 ^a^	202 ± 3 ^a^
	Wine *R*	397 ± 7 ^b^	396 ± 5 ^b^	325 ± 5 ^b^	314 ± 6 ^b^	257 ± 5 ^b^
F-A^+^ products ^2^	Wine *C*	0.97 ± 0.08 ^a^	1.01 ± 0.09 ^a^	1.31 ± 0.01 ^a^	0.28 ± 0.01 ^a^	0.17 ± 0.01 ^a^
	Wine *R*	0.98 ± 0.09 ^a^	1.15 ± 0.08 ^b^	1.39 ± 0.02 ^b^	0.32 ± 0.01 ^b^	0.22 ± 0.03 ^b^
F-ethyl-A^+^ products ^3^	Wine *C*	0.27 ± 0.02 ^a^	0.21 ± 0.01 ^a^	0.12 ± 0.01 ^a^	n.q.	n.q.
	Wine *R*	0.38 ± 0.01 ^b^	0.33 ± 0.02 ^b^	0.2 ± 0.01 ^b^	n.q.	n.q.
A-type + B-type vitisins of malvidin 3-*O*-glucoside	Wine *C*	3.43 ± 0.05 ^a^	2.85 ± 0.17 ^a^	1.72 ± 0.08 ^a^	1.45 ± 0.09 ^a^	1.59 ± 0.01 ^a^
	Wine *R*	3.51 ± 0.07 ^a^	3.04 ± 0.12 ^b^	1.73 ± 0.06 ^a^	1.47 ± 0.06 ^a^	1.74 ± 0.03 ^b^
Vinylphenol piranoanthocyanin of malvidin 3-*O*-glucoside	Wine *C*	0.06 ± 0.01 ^a^	0.09 ± 0.02 ^a^	0.1 ± 0.03 ^a^	0.06 ± 0.02 ^a^	0.14 ± 0.05 ^a^
	Wine *R*	0.05 ± 0.02 ^a^	0.09 ± 0.02 ^a^	0.1 ± 0.02 ^a^	0.07 ± 0.02 ^a^	0.18 ± 0.02 ^a^

^1^ Different lower case letters within each column and for each type of pigment indicate significant differences (*n* = 3, *p* < 0.05). ^2^ F-A^+^ products: Flavanol-anthocyanin direct condensation products. ^3^ F-ethyl-A^+^ products: Flavanol-anthocyanin acetaldehyde-mediated condensation products. n.q.: non-quantifiable.

**Table 5:** Evolution of CIELAB color parameters during winemaking and aging of Wines *C* (reference wine) and *R* (added with enological tannin).

		Sampling Point ^1^				
		17 Days	20 Days	2 Months	5 Months	8 Months
L*	Wine *C*	63.76 ± 0.04 ^a^	66.7 ± 0.09 ^a^	79.8 ± 0.07 ^a^	85.95 ± 0.04 ^a^	87.68 ± 0.03 ^a^
	Wine *R*	61.44 ± 0.04 ^b^	61.66 ± 0.02^b^	76.21 ± 0.01 ^b^	84.12 ± 0.03 ^b^	82.98 ± 0.01 ^b^
C*_ab_	Wine *C*	45.93 ± 0.05 ^a^	40.76 ± 0.09 ^a^	23.33 ± 0.06 ^a^	14.43 ± 0.07 ^a^	11.76 ± 0.01 ^a^
	Wine *R*	47.58 ± 0.07 ^b^	46.05 ± 0.03 ^b^	26.79 ± 0.02 ^b^	15.39 ± 0.06 ^b^	16.53 ± 0.01 ^b^
h_ab_	Wine *C*	−7.81 ± 0.03 ^a^	−7.26 ± 0.04 ^a^	−5.89 ± 0.05 ^a^	2.58 ± 0.04 ^a^	11.32 ± 0.14 ^a^
	Wine *R*	−7.13 ± 0.03 ^b^	−6.49 ± 0.02 ^b^	−5.91 ± 0.05 ^b^	6.79 ± 0.06 ^b^	7.97 ± 0.02 ^b^

^1^ Different lower case letters within each column and for each color parameter (L*, C*_ab_ or h_ab_) indicate significant differences (*n* = 3, *p* < 0.05).

## Supplementary Materials

The following are available online; **Grapes and wines:** This section reports the detailed information about the grapes and the winemaking procedure employed. **Table S1.** Evolution of the percentage in relation to the initial content of the different anthocyanin 3-*O*-glucosides in the model systems prepared in standard wine; **Table S2.** Evolution of the percentage in relation to the initial content of the different anthocyanin 3-*O*-glucosides in the model systems prepared in the fermentative medium.

## References

[1] He F., Liang N.N., Mu L., Pan Q.H., Wang J., Reeves M.J., Duan C.Q. (2012). Anthocyanins and their variation in red wines II. Anthocyanin derived pigments and their color evolution. Molecules.

[2] Salas E., Le Guerneve C., Fulcrand H., Poncet-Legrand C., Cheynier W. (2004). Structure determination and colour properties of a new directly linked flavanol-anthocyanin dimer. Tetrahedron Lett..

[3] Escribano-Bailón T., Álvarez-García M., Rivas-Gonzalo J.C., Heredia F.J., Santos-Buelga C. (2001). Color and stability of pigments derived from the acetaldehyde-mediated condensation between malvidin 3-O-glucoside and (+)-catechin. J. Agric. Food Chem..

[4] Fulcrand H., Benabdeljalil C., Rigaud J., Cheynier V., Moutounet M. (1998). A new class of wine pigments generated by reaction between pyruvic acid and grape anthocyanins. Phytochemistry.

[5] Fulcrand H., dosSantos P.J.C., SarniManchado P., Cheynier V., FavreBonvin J. (1996). Structure of new anthocyanin-derived wine pigments. J. Chem. Soc. Perkin Trans..

[6] Bakker J., Timberlake C.F. (1997). Isolation, identification, and characterization of new color-stable anthocyanins occurring in some red wines. J. Agric. Food Chem..

[7] Benabdeljalil C., Cheynier V., Fulcrand H., Hakiki A., Mosaddak M., Moutounet M. (2000). Evidence of new pigments resulting from reaction between anthocyanins and yeast metabolites. Sci. Aliment..

[8] Quijada-Morín N., Dangles O., Rivas-Gonzalo J.C., Escribano-Bailón M.T. (2010). Physico-chemical and chromatic characterization of malvidin 3-glucoside-vinylcatechol and malvidin 3-glucoside-vinylguaiacol wine pigments. J. Agric. Food Chem..

[9] Hayasaka Y., Asenstorfer R.E. (2002). Screening for potential pigments derived from anthocyanins in red wine using nanoelectrospray tandem mass spectrometry. J. Agric. Food Chem..

[10] Håkansson A.E., Pardon K., Hayasaka Y., de Sa M., Herderich M. (2003). Structures and colour properties of new red wine pigments. Tetrahedron Lett..

[11] Schwarz M., Wabnitz T.C., Winterhalter P. (2003). Pathway leading to the formation of anthocyanin-vinylphenol adducts and related pigments in red wines. J. Agric. Food Chem..

[12] Francia-Aricha E.M., Guerra M.T., Rivas-Gonzalo J.C., Santos-Buelga C. (1997). New anthocyanin pigments formed after condensation with flavanols. J. Agric. Food Chem..

[13] Rivas-Gonzalo J.C., Bravo-Haro S., Santos-Buelga C. (1995). Detection of compounds formed through the reaction of malvidin 3-monoglucoside and catechin in the presence of acetaldehyde. J. Agric. Food Chem..

[14] Blanco-Vega D., López-Bellido F.J., Alia-Robledo J.M., Hermosin-Gutierrez I. (2011). HPLC-DAD-ESI-MS/MS Characterization of Pyranoanthocyanins Pigments Formed in Model Wine. J. Agric. Food Chem..

[15] Mattivi F., Guzzon R., Vrhovsek U., Stefanini M., Velasco R. (2006). Metabolite profiling of grape: Flavonols and anthocyanins. J. Agric. Food Chem..

[16] García-Estevéz I., Alcalde-Eon C., Escribano-Bailón M.T. (2017). Flavanol Quantification of Grapes via Multiple Reaction Monitoring Mass Spectrometry: Application to Differentiation among Clones of *Vitis vinifera* L. cv. Rufete Grapes. J. Agric. Food Chem..

[17] Suárez-Lepe J.A., Morata A. (2012). New trends in yeast selection for winemaking. Trends Food Sci. Technol..

[18] Vivas N., Glories Y. (1996). Role of oak wood ellagitannins in the oxidation process of red wines during aging. Am. J. Enol. Vitic..

[19] Timberlake C.F., Bridle P. (1976). Interactions between anthocyanins, phenolic compounds, and acetalheyde and their significance in red wines. Am. J. Enol. Vitic..

[20] García-Estévez I., Escribano-Bailón M.T., Rivas-Gonzalo J.C., Alcalde-Eon C. (2012). Validation of a Mass Spectrometry Method to Quantify Oak Ellagitannins in Wine Samples. J. Agric. Food Chem..

[21] Cadahía E., Varea S., Muñoz L., Fernández de Simón B., García-Vallejo M.C. (2001). Evolution of ellagitannins in Spanish, French, and American oak woods during natural seasoning and toasting. J. Agric. Food Chem..

[22] Versari A., du Toit W., Parpinello G.P. (2013). Oenological tannins: A review. Aust. J. Grape Wine Res..

[23] Obreque-Slier E., Peña-Neira A., López-Solis R., Ramírez-Escudero C., Zamora-Marín F. (2009). Phenolic characterization of commercial enological tannins. Eur. Food Res. Technol..

[24] Bautista-Ortín A.B., Fernández-Fernández J.I., López-Roca J.M., Gómez-Plaza E. (2007). The effects of enological practices in anthocyanins, phenolic compounds and wine colour and their dependence on grape characteristics. J. Food Comp. Anal..

[25] Manfroi V., Rizzon L.A., Guerra C.C., Fialho F.B., Dall’Agnol I., Carlos Ferri V., Valmor Rombaldi C. (2010). Influence of different doses and distinct times of application of Enological tannins on the physicochemical characteristics of the Cabernet Sauvignon wine. Ciencia Tecnol. Aliment..

[26] Neves A.C., Spranger M.I., Zhao Y., Leandro M.C., Sun B. (2010). Effect of Addition of Commercial Grape Seed Tannins on Phenolic Composition, Chromatic Characteristics, and Antioxidant Activity of Red Wine. J. Agric. Food Chem..

[27] Liu Y.-X., Liang N.-N., Wang J., Pan Q.-H., Duan C.-Q. (2013). Effect of the Prefermentative Addition of Five Enological Tannins on Anthocyanins and Color in Red Wines. J. Food Sci..

[28] Parker M., Smith P.A., Birse M., Farancis I.L., Kwiatkowski M.J., Lattey K.A., Liebich B., Herderich M.J. (2007). The effect of pre- and post-ferment additions of grape derived tannin on Shiraz wine sensory properties and phenolic composition. Aust. J. Grape Wine Res..

[29] Main G.L., Morris J.R. (2007). Effect of macerating enzymes and postfermentation grape-seed tannin on the color of cynthiana wines. Am. J. Enol. Vitic..

[30] Alcalde-Eon C., García-Estévez I., Ferreras-Charro R., Rivas-Gonzalo J.C., Ferrer-Gallego R., Escribano-Bailón M.T. (2014). Adding oenological tannin vs. overripe grapes: Effect on the phenolic composition of red wines. J. Food Comp. Anal..

[31] Alcalde-Eon C., García-Estévez I., Puente V., Rivas-Gonzalo J.C., Escribano-Bailón M.T. (2014). Color stabilization of red wines. A chemical and colloidal approach. J. Agric. Food Chem..

[32] Asenstorfer R.E., Markides A.J., Iland P.G., Jones G.P. (2003). Formation of vitisin A during red wine fermentation and maturation. Aust. J. Grape Wine Res..

[33] García-Estévez I., Rivas-Gonzalo J.C., Escribano-Bailón M.T., Alcalde-Eon C. Evaluation of the influence of oak ellagitannins in the synthesis of pyranoanthocyanins in model systems. Proceedings of the 8th International Workshop on Anthocyanins (IWA).

[34] García-Puente Rivas E., Alcalde-Eon C., Santos-Buelga C., Rivas-Gonzalo J.C., Escribano-Bailón M.T. (2006). Behaviour and characterisation of the colour during red wine making and maturation. Anal. Chim. Acta.

[35] González-Manzano S., Dueñas M., Rivas-Gonzalo J.C., Escribano-Bailón M.T., Santos-Buelga C. (2009). Studies on the copigmentation between anthocyanins and flavan-3-ols and their influence in the colour expression of red wine. Food Chem..

[36] Boulton R. (2001). The copigmentation of anthocyanins and its role in the color of red wine: A critical review. Am. J. Enol. Vitic..

[37] Darias-Martín J., Carrillo M., Díaz E., Boulton R.B. (2001). Enhancement of red wine colour by pre-fermentation addition of copigments. Food Chem..

[38] Alcalde-Eon C., Escribano-Bailón M.T., Santos-Buelga C., Rivas-Gonzalo J.C. (2006). Changes in the detailed pigment composition of red wine during maturity and ageing—A comprehensive study. Anal. Chim. Acta.

[39] García-Estévez I., Jacquet R., Alcalde-Eon C., Rivas-Gonzalo J.C., Escribano-Bailón M.T., Quideau S. (2013). Hemisynthesis and Structural and Chromatic Characterization of Delphinidin 3-O-Glucoside-Vescalagin Hybrid Pigments. J. Agric. Food Chem..

[40] García-Marino M., Rivas-Gonzalo J.C., Ibáñez E., García-Moreno C. (2006). Recovery of catechins and proanthocyanidins from winery by-products using subcritical water extraction. Anal. Chim. Acta.

[41] Ough C.S., Davenport M., Joseph K. (1989). Effects of certain vitamins on growth and fermentation rate of several commercial active dry wine yeasts. Am. J. Enol. Vitic..

