# Supplementary material for: Enological Tannin Effect on Red Wine Color and Pigment Composition and Relevance of the Yeast Fermentation Products
Source: Molecules. 2017 Nov 23;22(12):2046. doi: 10.3390/molecules22122046 (PMC6149749; doi:10.3390/molecules22122046)
Supplement: Supplementary file 1 [file molecules-22-02046-s001.pdf]

Article

Enological Tannin Effect on Red Wine Color and Pigment Composition and Relevance of the Yeast Fermentation Products

Ignacio García-Estévez [1], **Cristina** Alcalde-Eon [1], **Víctor** Puente [2], **M. Teresa** Escribano-Bailón [1,*]

[1] Grupo de Investigacion en Polifenoles, Departament of Analytical Chemistry, Nutrition and Food Science, Faculty of Pharmacy, University of Salamanca, Campus Miguel de Unamuno, E 37007 Salamanca, Spain; igarest@usal.es (I.G.-E.), crisalcaldeon@usal.es (C.A.-E.)

[2] Laffort España, Polígono Txirrita Maleo 12, E 20100, Errenteria, Spain; victor.puente@laffort.com

* Correspondence: escriban@usal.es; Tel.: +34-923-294537

Grapes and wines

All wines studied were elaborated from the same "batch" of *Vitis vinifera L.* cv. Tempranillo grapes, which were collected the same day at technological maturity (24 ˚Brix) from a vineyard located in the D.O.Ca. Rioja. Grape juice showed 4.9 g/L of titrable acidity (expressed as tartaric acid equivalents), 3.4 g/L of malic acid, pH=3.93 and IPT=34. Fermentation was carried out with *S. cerevisiae* (*Zymaflore RJA64*, Laffort España, Rentería, Spain). Winemaking was performed in 100L stainless steel fermenters. Grape juice underwent cold macerations for 3 days, then it underwent alcoholic fermentation for 16 days and finally, wines underwent post-fermentative maceration for 3 days. SO_2 was added before (5g/hL) and after (4g/hL) alcoholic fermentation and temperature was controlled to be lower than 13 ºC, 23 ºC and 20ºC during cold maceration, fermentation process and post-fermentative maceration, respectively. Furthermore, during fermentation process the cap was punched down each two days. After post-fermentative maceration, malolactic fermentation was induced by inoculation with lactic acid bacteria (*Oenococcus oeni*).

R and C wines were elaborated following the same procedure but in the case of R wines, the enological tannin *C* was added at the start (10 g/hL), during the first *pumping over* (15 g/hL) and at the end of the alcoholic fermentation (10 g/hL) to reach a final concentration of 35 g/hL, as the manufacturer recommends.

Supplementary Materials

Table S1. Evolution of the percentage in relation to the initial content of the different anthocyanin 3-*O*-glucoside in the model systems prepared in standard wine.

	**Percentage in relation to initial content***						
		**Dp 3-glc****				**Cy 3-glc****	
**Day**	*ATC*	*ATS*	*A*	**Day**	*ATC*	*ATS*	*A*
0	100.00^{a}	100.00^{a}	100.00^{a}	0	100.00^{a}	100.00^{a}	100.00^{a}
5	77.23^{a}	81.69^{a}	82.11^{a}	5	88.53^{a}	89.44^{a}	89.50^{a}
8	63.90^{a}	63.46^{a}	65.78^{a}	8	83.51^{a}	83.24^{a}	83.42^{a}
11	56.40^{a}	58.34$^{a.b}$	59.99^{b}	11	79.14^{a}	79.50^{a}	80.46^{a}
19	30.61^{a}	31.58$^{a.b}$	32.98^{a}	19	64.35^{a}	62.98^{a}	63.64^{a}
26	18.08^{a}	19.26^{b}	20.61^{c}	26	55.86^{a}	55.89^{a}	56.58^{a}
33	10.34^{a}	11.46^{a}	12.87^{b}	33	48.92^{a}	49.34^{a}	50.85^{a}
41	4.45^{a}	5.52^{b}	6.63^{c}	41	40.82^{a}	41.48$^{a.b}$	43.66^{b}
49	2.28^{a}	2.81^{b}	3.32^{c}	49	35.34^{a}	35.98^{a}	37.07^{b}
56	1.10^{a}	1.36^{b}	1.62^{c}	56	30.57^{a}	30.67^{a}	32.44^{b}
63	0.48^{a}	0.64^{b}	0.76^{b}	63	24.35^{a}	24.39^{a}	26.17^{b}
70	0.23^{a}	0.27$^{a.b}$	0.32^{b}	70	18.63^{a}	18.34^{a}	20.18^{b}
78	0.19^{a}	0.22^{a}	0.24^{a}	78	18.25^{a}	18.25^{a}	20.94^{b}
90	n.d.	n.d.	n.d.	90	12.63^{a}	12.62^{a}	13.94^{b}
98	n.d.	n.d.	n.d.	98	11.31^{a}	10.92^{b}	12.40^{c}
**Day**		**Pt 3-glc****		**Day**		**Pn 3-glc****	
0	100.00^{a}	100.00^{a}	100.00^{a}	0	100.00^{a}	100.00^{a}	100.00^{a}
5	84.73^{a}	86.38^{a}	87.79^{a}	5	88.40^{a}	89.70^{a}	90.84^{a}
8	77.72^{a}	77.24^{a}	78.56^{a}	8	84.40^{a}	83.53^{a}	84.26^{a}
11	72.10^{a}	72.74^{a}	74.83^{a}	11	80.71^{a}	80.83^{a}	82.59^{a}
19	52.47^{a}	52.84^{a}	54.65^{a}	19	70.02^{a}	69.81^{a}	70.69^{a}
26	41.65^{a}	42.52^{a}	44.40^{b}	26	62.33^{a}	62.40^{a}	63.65^{b}
33	32.29^{a}	33.84$^{a.b}$	35.97^{b}	33	56.70^{a}	56.07^{a}	58.31^{b}
41	23.16^{a}	24.53^{a}	26.68^{b}	41	49.53^{a}	48.56^{a}	51.68^{b}
49	12.92^{a}	13.95^{b}	15.28^{c}	49	44.53^{a}	43.61^{a}	46.41^{b}
56	8.88^{a}	9.60^{a}	11.14^{b}	56	39.69^{a}	38.61^{a}	41.58^{b}
63	4.87^{a}	5.82^{b}	6.56^{c}	63	33.86^{a}	33.88^{a}	36.56^{b}
70	2.87^{a}	3.42^{b}	4.11^{c}	70	30.74^{a}	30.05^{a}	32.78^{b}
78	2.06^{a}	2.45$^{a.b}$	3.12^{b}	78	28.07^{a}	26.85^{a}	30.10^{b}
90	n.d.	n.d.	n.d.	90	23.19^{a}	22.13^{b}	24.70^{c}
98	n.d.	n.d.	n.d.	98	22.19^{a}	21.08^{b}	24.25^{c}
**Day**		**Mv 3- glc****		**Day**		**Total anthocyanin 3-glc****	
0	100.00^{a}	100.00^{a}	100.00^{a}	0	100.00^{a}	100.00^{a}	100.00^{a}
5	87.13^{a}	87.72^{a}	89.10^{a}	5	85.28^{a}	86.88^{a}	87.98^{a}
8	85.43^{a}	84.62^{a}	85.77^{a}	8	80.02^{a}	79.37^{a}	80.62^{a}
11	80.33^{a}	80.40^{a}	82.38^{a}	11	74.71^{a}	75.25^{a}	77.08^{a}
19	66.69^{a}	66.59^{a}	68.42^{a}	19	58.39^{a}	58.51^{a}	60.00^{b}
26	61.94^{a}	61.76^{a}	64.32^{b}	26	50.49^{a}	50.84^{a}	52.70^{b}
33	56.23^{a}	56.52^{a}	59.10^{b}	33	43.75^{a}	44.29^{a}	46.44^{b}
41	49.60^{a}	49.47^{a}	52.53^{b}	41	36.65^{a}	36.93^{a}	39.42^{b}
49	44.41^{a}	44.24^{a}	47.32^{b}	49	31.15^{a}	31.26^{a}	33.41^{b}
56	39.71^{a}	39.19^{a}	42.39^{b}	56	27.16^{a}	26.96^{a}	29.22^{b}
63	31.62^{a}	31.28^{a}	34.18^{b}	63	21.60^{a}	21.69^{a}	23.61^{b}
70	27.76^{a}	27.27^{a}	30.26^{b}	70	18.73^{a}	18.53^{a}	20.48^{b}
78	27.66^{a}	26.62^{a}	30.05^{b}	78	18.03^{a}	17.48^{a}	19.75^{b}
90	18.97^{a}	18.63^{a}	21.20^{b}	90	12.84^{a}	12.52^{a}	14.12^{b}
98	17.68^{a}	17.00^{b}	19.65^{c}	98	12.05^{a}	11.56^{b}	13.32^{c}

* **Dp**: delphinidin. **Cy**: Cyanidin. **Pt**: Petunidin: **Pn**: Peonidin. **Mv**: Malvidin. *AC*: Model system added with tannin *C*. *AS*: Model system added with tannin *S*. *A*: Reference model system.

** Different lower case letters within each file and for each anthocyanin indicate significant differences (n=6. p<0.05). n.d.: non-detected.

Table S2. Evolution of the percentage in relation to the initial content of the different anthocyanin 3-*O*-glucoside in the model systems prepared in the fermentative medium.

Percentage in relation to initial content*

Dp 3-glc**

Day	*ATCF*	*ATSF*	*AF*
0	100.00^{a}	100.00^{a}	100.00a
5	67.70^{a}	65.27^{a}	63.09^{a}
8	40.57^{a}	39.56^{a}	34.83^{b}
11	32.40^{a}	30.89^{a}	26.55^{b}
19	8.66^{a}	8.03^{a}	5.37^{b}
26	2.43^{a}	2.32^{a}	1.25^{b}
33	0.86^{a}	0.81^{a}	0.47^{b}
41	0.31^{a}	0.29^{a}	0.19^{b}
49	0.16^{a}	0.16^{a}	n.d.
56	n.d.	n.d.	n.d.
63	n.d.	n.d.	n.d.
70	n.d.	n.d.	n.d.
78	n.d.	n.d.	n.d.
90	n.d.	n.d.	n.d.
98	n.d.	n.d.	n.d.

Cy 3-glc**

Day	*ATCF*	*ATSF*	*AF*
0	100.00^{a}	100.00^{a}	100.00^{a}
5	79.51^{a}	79.16^{a}	80.98^{a}
8	66.33^{a}	66.53^{a}	66.60^{a}
11	60.98^{a}	60.44^{a}	60.36^{a}
19	39.67^{a}	39.24^{a}	38.51^{b}
26	28.87^{a}	28.36^{a}	26.61^{b}
33	21.69^{a}	21.20^{a}	19.59^{b}
41	15.21^{a}	14.33^{a}	13.01^{b}
49	10.26^{a}	10.07^{a}	9.46^{b}
56	7.35^{a}	7.32^{a}	6.22^{b}
63	5.26^{a}	5.07^{a}	4.05^{b}
70	3.86^{a}	3.71^{a}	2.83^{b}
78	2.60^{a}	2.61^{a}	1.84^{b}
90	1.53^{a}	1.45^{a}	1.02^{b}
98	1.20^{a}	1.11^{a}	0.80^{b}

Pt 3-glc**

Day	*ATCF*	*ATSF*	*AF*
0	100.00^{a}	100.00^{a}	100.00a
5	76.11^{a}	74.19^{a}	74.98^{a}
8	55.52^{a}	55.47^{a}	53.50^{a}
11	49.54^{a}	48.03^{a}	45.97^{b}
19	23.56^{a}	22.71^{a}	19.79^{b}
26	10.07^{a}	9.53^{a}	6.54^{b}
33	4.72^{a}	4.37^{a}	2.55^{b}
41	1.67^{a}	1.59^{a}	0.74^{b}
49	0.66^{a}	0.69^{a}	0.35^{b}
56	0.27^{a}	0.30^{a}	0.16^{b}
63	n.d.	n.d.	n.d.
70	n.d.	n.d.	n.d.
78	n.d.	n.d.	n.d.
90	n.d.	n.d.	n.d.
98	n.d.	n.d.	n.d.

Pn 3-glc**

Day	*ATCF*	*ATSF*	*AF*
0	100.00^{a}	100.00^{a}	100.00^{a}
5	83.28^{a}	82.79^{a}	86.11^{a}
8	72.73^{a}	72.56^{a}	74.90^{a}
11	67.99^{a}	67.39^{a}	69.66^{a}
19	49.64^{a}	49.03^{a}	50.83^{a}
26	39.54^{a}	38.52^{a}	39.29^{a}
33	31.59^{a}	30.71^{a}	31.25^{a}
41	24.37^{a}	23.25^{a}	23.73^{a}
49	19.56^{a}	18.53^{b}	19.40^{a}
56	15.29^{a}	14.30^{b}	14.40^{b}
63	11.26^{a}	10.36^{b}	10.44^{b}
70	8.86^{a}	8.04^{b}	8.18^{b}
78	6.97^{a}	6.14^{b}	6.23^{b}
90	4.67^{a}	4.09^{b}	4.07^{b}
98	3.71^{a}	3.21^{b}	3.17^{b}

Mv 3-glc*

Day	*ATCF*	*ATSF*	*AF*
0	100.00^{a}	100.00^{a}	100.00^{a}
5	80.81^{a}	79.82^{a}	83.21^{a}
8	70.04^{a}	69.70^{a}	72.24^{a}
11	64.61^{a}	63.99^{a}	66.48^{a}
19	44.63^{a}	43.97^{a}	46.36^{a}
26	35.65^{a}	34.65^{a}	35.60^{a}
33	28.08^{a}	27.89^{a}	27.92^{a}
41	21.15^{a}	21.00^{a}	20.76^{a}
49	16.70^{a}	15.84^{a}	16.91^{a}
56	12.77^{a}	12.44^{a}	11.81^{b}
63	9.16^{a}	9.20^{a}	8.35^{b}
70	6.97^{a}	6.78^{a}	6.35^{b}
78	5.47^{a}	5.30^{a}	4.72^{b}
90	3.28^{a}	3.00^{a}	2.76^{b}
98	2.56^{a}	2.37^{a}	2.14^{b}

Total anthocyanin 3-glc**

Day	*ATCF*	*ATSF*	*AF*
0	100.00^{a}	100.00^{a}	100.00^{a}
5	78.06^{a}	76.77^{a}	78.63^{a}
8	62.63^{a}	62.27^{a}	62.58^{a}
11	56.74^{a}	55.82^{a}	56.14^{a}
19	35.31^{a}	34.63^{a}	34.95^{a}
26	25.77^{a}	25.31^{a}	24.76^{b}
33	19.60^{a}	19.36^{a}	18.90^{b}
41	14.43^{a}	14.26^{a}	13.84^{b}
49	11.24^{a}	11.17^{a}	11.16^{a}
56	8.57^{a}	8.43^{a}	8.04^{b}
63	6.17^{a}	6.01^{a}	5.61^{b}
70	4.74^{a}	4.63^{a}	4.29^{b}
78	3.70^{a}	3.56^{a}	3.20^{b}
90	2.31^{a}	2.23^{a}	1.95^{b}
98	1.81^{a}	1.79^{a}	1.01^{b}

* **Dp**: delphinidin. **Cy**: Cyanidin. **Pt**: Petunidin: **Pn**: Peonidin. **Mv**: Malvidin. *ACF*: Model system added with tannin *C*. *ASF*: Model system added with tannin *S*. *AF*: Reference model system.

** Different lower case letters within each file and for each anthocyanin indicate significant differences (n=6. $p<0.05$). n.d.: non-detected.
